# Novel microbiota *Mesosutterella faecium* sp. nov. has a protective effect against inflammatory bowel disease

**DOI:** 10.3389/fmicb.2024.1342098

**Published:** 2024-04-02

**Authors:** Seung Yeob Yu, Byeong Seob Oh, Seoung Woo Ryu, Jeong Eun Bak, Eun Seo Heo, Jeong Chan Moon, Jae-Ho Jeong, Ju Huck Lee

**Affiliations:** ^1^Korean Collection for Type Cultures, Biological Resource Center, Korea Research Institute of Bioscience and Biotechnology, Jeongeup, Republic of Korea; ^2^BioMedical Sciences Graduate Program (BMSGP), Chonnam National University, Hwasun, Republic of Korea; ^3^University of Science and Technology (UST), Daejeon, Republic of Korea; ^4^National Institute of Ecology, Yeongyang, Republic of Korea

**Keywords:** gut microbiota, *Mesosutterella*, inflammation, inflammatory bowel disease, murine colitis model

## Abstract

A novel Gram-negative, obligate anaerobe, non-motile, flagella-lacking, catalase- and oxidase-negative, coccobacilli-shaped bacterial strain designated AGMB02718^T^ was isolated from swine feces. The 16S rRNA gene analysis indicated that strain AGMB02718^T^ belonged to the genus *Mesosutterella* with the highest similarity to *M. multiformis* 4NBBH2^T^ (= DSM 106860^T^) (sequence similarity of 96.2%), forming a distinct phylogenetic lineage. Its growth occurred at 25–45°C (optimal 37°C) and in 0.5–1% NaCl (optimal 0.5%). Strain AGMB02718^T^ was asaccharolytic and contained menaquinone 6 (MK-6) and methylmenaquinone 6 (MMK-6) as the predominant respiratory quinones. The major cellular fatty acids in the isolate were C_18:1_*ω*9*c* and C_16:0_. Based on the whole-genome sequencing analysis, strain AGMB02718^T^ had a 2,606,253 bp circular chromosome with a G + C content of 62.2%. The average nucleotide identity value between strain AGMB02718^T^ and *M. multiformis* 4NBBH2^T^ was 72.1%, while the digital DNA–DNA hybridization value was 20.9%. Interestingly, genome analysis suggested that strain AGMB02718^T^ possessed a low-toxicity lipopolysaccharide (LPS) because the genome of the isolate does not include *lpxJ* and *lpxM* genes for Kdo_2_-Lipid A (KLA) assembly, which confers high toxicity to LPS. Moreover, *in vitro* macrophage stimulation assay confirmed that AGMB02718^T^ produced LPS with low toxicity. Because the low-toxicity LPS produced by the *Sutterellaceae* family is involved in regulating host immunity and low-toxicity LPS-producing strains can help maintain host immune homeostasis, we evaluated the anti-inflammatory activity of strain AGMB02718^T^ against inflammatory bowel disease (IBD). As a result, strain AGMB02718^T^ was able to prevent the inflammatory response in a dextran sulfate sodium (DSS)-induced colitis model. Therefore, this strain represents a novel species of *Mesosutterella* that has a protective effect against DSS-induced colitis, and the proposed name is *Mesosutterella faecium* sp. nov. The type strain is AGMB02718^T^ (=GDMCC 1.2717^T^ = KCTC 25541^T^).

## 1 Introduction

The genus *Mesosutterella* comprises obligate anaerobe, Gram-negative, coccobacilli. The first member of the genus, *M. multiformis*, was isolated from the fecal samples of a healthy Japanese woman and man (Sakamoto et al., 2018). *Mesosutterella* was classified as a neighboring branch of the genera *Parasutterella* and *Sutterella* by Sakamoto et al. (2018). The genus *Mesosutterella*, along with the genera *Sutterella* and *Parasutterella*, belongs to the family *Sutterellaceae* and is distinguished from *Sutterella* by its fatty acid production (Sakamoto et al., 2018). Members of the family *Sutterellaceae* are mostly found in the intestinal tract of humans and some animals as members of the indigenous gut microbiota. They are microbes that adhere to the intestinal epithelial cells (IECs) and, because they are covered by mucus, they form an epithelial barrier between the luminal microbes and the mucus (Johansson et al., 2013; Pelaseyed et al., 2014; Donaldson et al., 2016). Previous studies have implicated this family member in inflammatory bowel disease (IBD) (Kaakoush, 2020; Gryaznova et al., 2021).

The gut microbiota is the microbial community that inhabits the gastrointestinal tract of an animal host. It is shaped by several environmental, dietary, and host-related factors and has a significant impact on the health of the host. Understanding the role of gut microbiota interventions in health has far-reaching implications for human and veterinary diseases (Jarett et al., 2021). Intestinal homeostasis is maintained through interactions between the balanced gut microbiota and the mucosal immune system; however, it can be easily perturbed by changes in the microbiome, resulting in a pro-inflammatory immune response. Dysbiosis plays an important role in the pathogenesis of IBD (Nishida et al., 2018), resulting in an imbalance between beneficial and harmful taxa and damage to the intestinal microbial barrier (Chelakkot et al., 2018), which not only occurs in humans but also in animals (Bomba et al., 2014; Honneffer et al., 2014). Epidemiological research revealed that the incidence of IBD is rapidly increasing worldwide, creating a global public health concern (GBD 2017 Inflammatory Bowel Disease Collaborators, 2020). IBD includes ulcerative colitis and Crohn’s disease, both of which are associated with chronic inflammation (Kobayashi et al., 2020). The expansion of bacteria that can potentiate the pathogenesis of IBD via lipopolysaccharide (LPS) induces severe inflammation in the host (Stephens and von der Weid, 2020). Microbe-associated molecular patterns (MAMPs) expressed by gut bacteria are recognized by various pattern recognition receptors in intestinal epithelial cells (IECs), including Toll-like receptors (TLRs) (Bron et al., 2011; Round et al., 2011). LPS, a typical MAMP produced by Gram-negative bacteria, triggers an inflammatory cascade by activating TLR4 signaling (Miller et al., 2005). The LPS of Gram-negative bacteria consists of Kdo_2_-Lipid A (KLA), a core-oligosaccharide, and O-antigen repeats, and is recognized by the host TLR4-MD2 complex (Polissi and Sperandeo, 2014). *Escherichia coli* lipid A is highly toxic because it is hexa-acylated through two phosphate groups and activates the TLR4-Mal-MyD88 pathway, whereas the penta-acylated or monophosphorylated form of lipid A activates the TRL4-TRAM-TRIF pathway, and its toxicity is approximately 100 times lower than that of the hexa-acylated form (Park et al., 2009; Herath et al., 2011; Han et al., 2013). *Sutterella* spp. lacking the *lpxL* and *lpxM* genes, responsible for adding secondary acyl chains (hexa-acylation), has LPS with low toxicity and modulates immunity through intestinal adhesion (Hiippala et al., 2016). Differences in gut microbial LPS structure play an important role in modulating host immunity (Vatanen et al., 2016; Di Lorenzo et al., 2020). In addition, adherent commensal bacteria producing LPS with low toxicity exert preventive and therapeutic effects against IBD by maintaining host immune homeostasis (Steimle et al., 2019).

Metagenomics is a culture-independent approach that directly targets the DNA in all environments. This sequencing approach has been instrumental in elucidating the bacterial composition of different environments and their effects on health and disease and has been widely used for comprehensive analysis of the gut microbiota (Martin et al., 2014). However, it has recently been suggested that sequencing-based metagenomics may have limitations in elucidating the function of the microbiota owing to biases during sequencing and bioinformatics processes, which may lead to errors in determining microbiota functions (Lagier et al., 2012). Most importantly, a sequencing-based study cannot provide bacteria, which is needed for further functional studies and microbiota development (Lagier et al., 2012). Therefore, culture-based studies, which have been employed to determine the microbiome composition before using metagenomics, have recently gained attention in the microbiome field because they can overcome the limitations of metagenomic analyses. Therefore, we attempted to isolate various gut microbiota from swine feces.

In the present study, a new strain, AGMB02718^T^, was isolated from swine feces while obtaining various kinds of gut microbes and the isolate was characterized as a novel species of the genus *Mesosutterella* based on phenotypic, phylogenetic, genotypic, and chemotactic analyses. It is noteworthy that because genome analysis showed that strain AGMB02718^T^ does not have *lpxJ* and *lpxM* genes, suggesting the potential of the isolate in preventing IBD, we examined its role *in vivo* using a DSS-induced colitis model. The results suggest the potential contribution of the *Mesosutterella faecium* AGMB02718^T^ strain, isolated from swine feces, to the prevention of IBD.

## 2 Materials and methods

### 2.1 Isolation of the bacterial strain and culture conditions

Strain AGMB02718^T^ was isolated from swine feces collected from a swine farm located in the National Institute of Animal Science in Cheonan, Republic of Korea (36.93 N, 127.11 E). To isolate gut microbiota, the fecal samples were placed in anaerobic chamber (Coy Laboratory Products, Grass Lake, MI, United States) in an atmosphere consisting of 86% N_2_, 7% CO_2_, and 7% H_2_. The stool was serially diluted to 10^−4^ in sterilized phosphate-buffered saline (PBS), and 100 μL of the diluted suspension was spread onto tryptic soy agar supplemented with 5% sheep blood (TSAB). After incubation for 72 h, single colonies were transferred to fresh TSAB. A convex, smooth, and circular colony of the strain AGMB02718^T^ was obtained and subjected to taxonomic analysis based on phenotypic, physiological, and phylogenetic characteristics. The isolate and reference strain, *M. multiformis* DSM 106860^T^, were routinely cultured on TSAB plates at 37°C in an anaerobic chamber for 72 h and preserved at − 80°C in 10% (w/v) skim milk. *E. coli* DH5α was grown overnight in LB medium at 37°C with aeration. AGMB02718^T^ was deposited in the Korean Collection for Type Cultures (KCTC) and Guangdong Microbial Culture Collection (GDMCC).

### 2.2 16S rRNA gene sequencing and phylogenetic analysis

For phylogenetic analysis, the 16S rRNA gene of strain AGMB02718^T^ was amplified by PCR from cell suspensions using the universal 16S rRNA bacterial primers 27F (5′-AGAGTTTGATCMTGGCTCAG-3′) and 1492R (5′-TACGGYTACCTTGTTACGACTT-3′). The amplified 16S rRNA gene was sequenced with the universal primers 518F (5′-CCAGCAGCCGCGGTAATACG-3′) and 805R (5′-GACTACCAGGGTATCTAATC-3′) (BIOFACT, Daejeon, Republic of Korea). The sequenced 16S rRNA gene was analyzed using BLAST searches in the EzBioCloud database (http://www.ezbiocloud.net; Yoon et al., 2017) and GenBank/EMBL/DDBJ databases to identify taxa related to strain AGMB02718^T^. Multiple alignments were performed using CLUSTAL W (Thompson et al., 1997) and gaps were edited using BioEdit (Hall, 1999). Phylogenetic trees were constructed using Molecular Evolutionary Genetics Analysis 11 (MEGA 11) software (Tamura et al., 2021) with different algorithms based on neighbor-joining (NJ) (Saitou and Nei, 1987), maximum likelihood (ML) (Fitch, 1971), and maximum parsimony (MP) (Kolaczkowski and Thornton, 2004) methods, performed using 1,000 bootstrap values. In addition, the evolutionary distances of all trees were determined using the Kimura-2-parameters method set to default.

### 2.3 Phenotypic and biochemical analyses

For the morphology analysis, strain AGMB02718^T^ was incubated anaerobically on TSAB plates for 72 h at 37°C. The cellular morphology was observed using phase-contrast microscopy (Eclipse 80i, Nikon, Tokyo, Japan), scanning electron microscopy (SEM; CX-200TA, Coxem, Daejeon, Republic of Korea), and transmission electron microscopy (TEM; CM-120, Philips, Amsterdam, Netherlands). For SEM analysis, the cultured cells were fixed with 4% paraformaldehyde and dehydrated with gradient ethanol solutions (10–100%). After fixation, samples were treated with isoamyl acetate and dried over hexamethyldisilazane (HMDS). Thereafter, the dried samples were coated with platinum using a sputter coater (SPT-20, Coxem, Daejeon, Republic of Korea) and observed by SEM. For the TEM analysis, the cells were fixed and applied to carbon-coated grids that had been glow-discharged for 3 min in air. Next, the grids were negatively stained with 1% uranyl acetate. Subsequently, the samples were observed using a TEM equipped with a lanthanum hexaboride cathode. Gram staining was performed using a commercial kit (Sigma-Aldrich, MA, United States) according to the manufacturer’s instructions. Various temperatures (15, 20, 25, 30, 37, 40, 45, and 50°C) were assayed to identify the growth range of the novel strain. The NaCl tolerance (0.5, 1, 2, 3, 4, and 5%, w/v) was measured on TSAB plates. The oxygen requirements for growth were examined by incubating strain AGMB02718^T^ under aerobic, microaerophilic (CO_2_ incubator filled with, 10% O_2_, 10% CO_2_, and 80% N_2_), and anaerobic (anaerobic chamber filled with 86% N_2_, 7% CO_2_, and 7% H_2_) conditions for 72 h at 37°C. The activities of catalase and oxidase were tested by using a commercial reagent (bioMérieux, Marcy-l’Étoile, France) following the manufacturer’s instructions. Other biochemical features, such as enzyme activities, utilization of substrates, and acid production from carbohydrates, were determined by using API 20A, API ZYM, and Rapid ID 32A strips according to the manufacturer’s protocols (bioMérieux, Marcy-l’Étoile, France). Closely related strain types were tested under the same conditions.

### 2.4 Chemotaxonomic characteristic

The cellular fatty acid compositions of the strain AGMB02718^T^ and the reference strain were examined. After harvesting the cells grown for 72 h at 37°C on TSAB plates, the whole cellular fatty acids were obtained through saponification, methylation, and extraction as detailed in the MIDI/Hewlett Packard Microbial Identification System (Sasser, 1990). The cellular fatty acids obtained were analyzed using gas chromatography (6,890 N with a 7683 autosampler; Agilent Technologies, California, United States). Fatty acids were identified using the Sherlock Microbial Identification System in the Anaerobe database v 6.1. Quinones were extracted from 100 mg of freeze-dried cells using chloroform:methanol (2:1; v/v) and used for respiratory quinone analysis. This analysis was performed according to the method described by Komagata and Suzuki (1988). The crude quinone compounds were purified by thin-layer chromatography (TLC) using petroleum benzene:diethyl ether (9:1; v/v) on a silica gel 60 F254 plate (20 × 20 mm, Merck, NJ, United States). The quinones were detected by reverse-phase HPLC with a mixture of methanol:isopropyl ether (3:1; v/v) as the mobile phase (flow rate of 0.7 mL min^−1^) by monitoring a UV detector at 270 nm.

### 2.5 Genomic analysis

Genomic DNA was extracted from AGMB02718^T^ using the phenol:chloroform:isoamyl alcohol method as described by Wilson et al. (1990). Whole-genome sequencing of the strain was performed using the PacBio Sequel System and Illumina NovaSeq technology, and filtered reads were assembled using Flye (ver. 2.9.2) and SPAdes (ver. 3.13.1) software, respectively. The assembled genome was annotated using the NCBI Prokaryotic Genome Annotation Pipeline (PGAP) (Tatusova et al., 2016). The predicted protein sequences were assigned to functional groups in the Clusters of Orthologous Groups (COG) using eggNOG 5.0. The metabolic pathways of strain AGMB02718^T^ were investigated using BlastKOALA, which is based on the Kyoto Encyclopedia of Genes and Genomes (KEGG) pathway database (Kanehisa et al., 2016). To analyze the genomic similarity between strain AGMB02718^T^ and the closely related taxa, the pairwise average nucleotide identity (ANI) values were calculated using ANI calculator^1^ and Orthologous Average Nucleotide Identity Tool (OAT) software (Lee et al., 2016), and digital DNA–DNA hybridization (dDDH) was calculated using the Genome-to-Genome Distance Calculator (GGDC) version 3.0 (https://ggdc.dsmz.de/ggdc.php#; Meier-Kolthoff et al., 2013). Additionally, phylogenomic tree was constructed based on the up-to-date bacterial core gene (UBCG) consisting of 92 single-copy core gene sets and pipelines, as described by Na et al. (2018). The available genomes of related taxa were retrieved from draft or complete genome sequences in the NCBI database. To construct the phylogenomic tree, UBCGs were extracted from a whole-genome assembly using Prodigal (Hyatt et al., 2010) and hmmsearch (Eddy, 2011). The extracted 92 core genes were aligned using multiple alignments with Fast Fourier Transform (MAFFT) (Katoh and Standley, 2013). A phylogenomic tree was generated using FastTree (Price et al., 2010) and viewed using MEGA 11. A circular map of the AGMB02718^T^ genome was constructed using CGView software.^2^

### 2.6 *In vitro* macrophage stimulation assay

The RAW264.7 cells were incubated in 24-well plates (3 × 10^5^ cells/well) with Dulbecco’s modified Eagle medium (DMEM, welgene, Gyeongsangbuk-do, Republic of Korea) supplemented with 10% fetal bovine serum and 1% antibiotic antimycotic solution (ABS, welgene, Gyeongsangbuk-do, Republic of Korea) for 24 h. Then, *E. coli* DH5α and AGMB02718^T^ were treated at a multiplicity of infection (MOI) of 0.1 (3 × 10^4^ CFU/well), 1 (3 × 10^5^ CFU/well), and 10 (3 × 10^6^ CFU/well) for 6 h. LPS was isolated from 1 × 10^9^ CFU of each bacterium using an LPS extraction kit (iNtRON Biotechnology, Gyeonggi-do, Republic of Korea) following the manufacturer’s instructions. The isolated LPS was subsequently treated at 0, 62.5, 125, and 250 μg/mL for 6 h, Afterward, the supernatant was collected for measurement. Cytokines (IL-6 and TNF-α) were quantified using ELISA (BD, NJ, United States), and the analysis was performed according to the manufacturer’s instructions.

### 2.7 Mice

Fifteen male C57BL/6 mice (5-week-old) were purchased from KOSA BIO (Seongnam, Republic of Korea). All mice were raised under specific pathogen-free conditions and housed with five heads per cage, allowing for spontaneous intake of food and water. Animals were kept under a 12 h light/dark cycle (light on 09:00–21:00) at room temperature (23 ± 2°C) and humidity (55 ± 10%). All mouse experiments were approved by the Institutional Animal Care and Use Committee of the Korea Research Institute of Bioscience and Biotechnology (approval number: KRIBB-AEC-22146) and all animals were cared for according to the guidelines for animal experiments of the Korea Research Institute of Bioscience and Biotechnology.

### 2.8 Induction of colitis and *Mesosutterella faecium* administration

A colitis model was established as described by Chassaing et al. (2014). After 1 week of acclimation, C57BL/6 mice were randomly divided into three groups (*n* = 5/group): control (PBS only), dextran sulfate sodium (DSS) (DSS and PBS treatment), and AGMB02718 (DSS and bacterial treatment). AGMB02718 group mice were administered *M. faecium* AGMB02718^T^ (1 × 10^8^ CFU/100 μL/mouse) once a day for 1 week. Colonic inflammation was induced by the addition of 2.5% (w/v) DSS (MW: 40,000; MP Biomedicals, CA, United States) to drinking water *ad libitum* for 10 days. DSS was administered 10 days after the start of PBS or bacterial administration. Body weight, stool consistency, and presence of gross rectal blood were evaluated daily. Weight change was calculated using the following formula:


$$\left[\frac{\mathrm{Weight}\;\mathrm{on}\;\mathrm{day}\;X-\mathrm{Initial}\;\mathrm{weight}}{\mathrm{Initial}\;\mathrm{weight}}\right]\times 100$$

The severity of colitis was calculated daily based on the Disease Activity Index (DAI) score, first described by Cooper et al. (1993). On the 10th day after the initiation of DSS treatment, all mice were sacrificed after collection of serum, feces, and colon tissue. Feces and serum were stored at −80°C until analysis. After measuring the length of the resected colon, it was segmented and stained with hematoxylin and eosin (H&E).

### 2.9 Histopathological analysis

The segmented colon tissue was fixed in 10% (v/v) neutral-buffered formalin (Sigma-Aldrich, MA, United States) and embedded in paraffin. The tissue was then cut into 5 μm thick sections and stained with H&E (BCC Biochemical, WA, United States). For histopathological analysis, at least three slides were randomly selected and graded in a blinded manner using a light microscope (Leica Microsystem, Wetzlar, Germany) with 4× and 20× objective lenses. The histological activity index (HAI) was measured based on the quality and dimensions of inflammatory cell infiltrates (1–5), epithelial changes (1–5), and overall mucosal structure (1–5) scores as previously described (Erben et al., 2014).

### 2.10 Intestinal permeability

Intestinal permeability was determined using fluorescein isothiocyanate-conjugated dextran (FITC-dextran; average MW 4000, Sigma-Aldrich, MA, United States) assays. Mice were fasted overnight and FITC-dextran (400 mg/kg) dissolved in PBS was administered by gavage 4 h before blood collection. The FITC-dextran concentration in the serum was measured at 485/538 nm using a microplate reader (Fluoroskan FL; Thermo Scientific, MA, United States).

### 2.11 Fecal microbial community analysis

Fecal samples were immediately frozen and stored at −80°C freezer after collection. Total bacterial DNA was extracted from fecal samples (approximately 200 mg) using a QIAamp Fast DNA Stool Mini Kit (Qiagen, Hilden, Germany), following the manufacturer’s instructions. The V3–V4 region of the bacterial 16S rRNA gene was amplified using 341F (5′-CCTACGGGNGGCWGCAG-3′) and 805R (5′-GACTACHVGGTATCTAATCC-3′) primers from each sample following standard procedures at an external facility (Macrogen, Seoul, South Korea). Identical sequencing reads were combined through DADA2’s dereplication functionality, and the DADA2 (v1.18.0) sequence–variant inference algorithm was applied to each dataset (Callahan et al., 2016). A taxonomy was assigned to each amplicon sequence variant (ASV) based on BLASTN (v2.9.0+) of the NCBI 16S Microbial DB (Zhang et al., 2000). For multiple alignments between representative sequences of ASVs, MAFFT (ver. 7.475) was used (Katoh and Standley, 2013). Alpha (α) diversity was applied to analyze the complexity of species diversity for a sample through ASVs, Chao1, and Shannon indexes. Beta (β) diversity group analysis was performed using a Jaccard index method, and divergence between the groups was highlighted by principal coordinates analysis (PCoA). α and β diversity were calculated with QIIME2 (ver. 1.9.0) (Caporaso et al., 2010). The feature abundance was normalized using the relative abundance of each sample. Functional predictions based on 16S rRNA gene data were performed using Tax4Fun provided by MicrobiomeAnalyst (Asshauer et al., 2015; Dhariwal et al., 2017).

## 3 Results and discussion

### 3.1 Phylogenetic analyses

**Figure 1:**
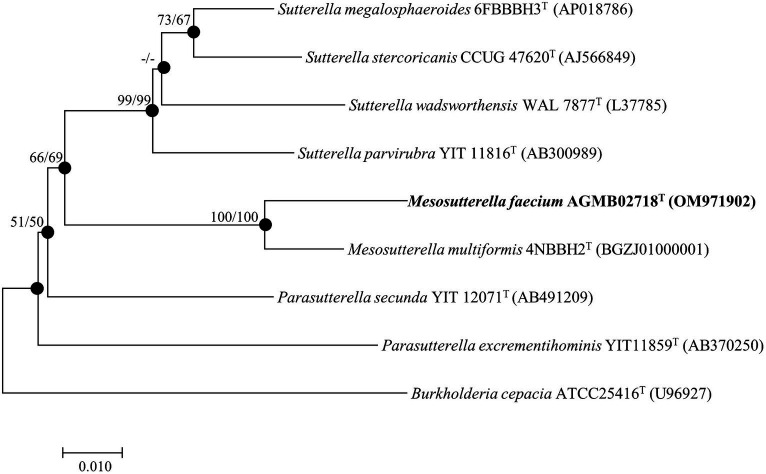
Neighbor-joining phylogenetic tree based on 16S rRNA gene sequences showing the phylogenetic position of strain AGMB02718^T^ and closely related taxa. Bootstrap values for NJ (left) and ML (right) are shown indicated at the nodes (based on 1,000 replicates, only values >50% are shown at branch points). Filled circles indicate that the corresponding nodes (groupings) were recovered by the neighbor-joining and maximum likelihood. Scale bar: 0.010 substitutions per nucleotide position.

The complete 16S rRNA gene sequence of strain AGMB02718^T^ contained 1,461 bp and was submitted to GenBank (accession number: OM971902). Comparative sequence analysis using the 16S rRNA gene sequences determined that strain AGMB02718^T^ belonged to the genus *Mesosutterella* and was closely related to *Mesosutterella multiformis* 4NBBH2^T^ (96.2%), *Sutterella stercoricanis* CCUG 47620^T^ (91.1%), *Sutterella megalosphaeroides* 6FBBBH3^T^ (91.0%), *Parasutterella secunda* YIT 12071^T^ (90.9%), *Sutterella wadsworthensis* WAL 7877^T^ (90.6%), and *Sutterella parvirubra* YIT 11816^T^ (90.4%). Based on the threshold of interspecies 16S rRNA gene sequence similarity percentages, which are between 95.0 and 98.7% (Beye et al., 2018), strain AGMB02718^T^ was considered a novel species in the genus *Mesosutterella*. The NJ, ML, and MP algorithms were used for phylogenetic tree analysis (Supplementary Figure S1). The phylogenetic tree reconstructed using NJ and ML algorithms revealed that strain AGMB02718^T^ formed a stable cluster with another strain of the genus *Mesosutterella* (Figure 1). The genera *Sutterella* and *Parasutterella* formed independent monophyletic branches. The phylogenetic results suggested that strain AGMB02718^T^ represents a novel species of the genus *Mesosutterella*. Therefore, *M. multiformis* DSM 106860^T^ was selected as the reference species for further comparative tests.

### 3.2 Morphological, phenotypic, and biochemical characteristics

Phenotypic characteristics based on the optimal growth medium, morphology, temperature, and salt tolerance were investigated. Strain AGMB02718^T^ grew well in the TSAB medium. Cells were obligate anaerobe, Gram-negative, coccobacilli-shaped with a mean width of 0.4 μm and mean length of 1.25 μm (Supplementary Figure S2). Growth of strain AGMB02718^T^ occurred in the range of 25–45°C (optimal 37°C). In addition, the isolate was able to grow in up to 1.0% (w/v) NaCl (optimal, 0.5%). The strain AGMB02718^T^ was negative for catalase and oxidase activities. The results are presented in Supplementary Table S1. When compared to the reference strain in API tests, including API 20A, API ZYM, and Rapid ID 32A, strain AGMB02718^T^ showed differences in salicin, D-raffinose, and D-sorbitol activities in API 20A, and differences in esterase (C4), cystine arylamidase, naphthol-AS-BI-phosphohydrolase, N-acetyl-b-glucosaminidase, glutamyl glutamic acid arylamidase, and serine arylamidase activities in API ZYM and Rapid ID32A. The different characteristics of strain AGMB02718^T^ compared with those of the reference strain are presented in Table 1. All API test results for AGMB02718^T^ and the reference strain are listed in Supplementary Table S2.

**Table 1:** Differential characteristics of strain AGMB02718^T^ and reference strain.

Characteristic	1	2
Isolation source	Swine feces	Human feces^†^
Morphology	Coccobacilli	Straight rods or coccobacilli^†^
Growth temperature range (°C, optimum)	25–45 (37)	20–45 (37)^†^
Cell size (μm)	0.4 × (1.0–1.5)	0.8 × (1.7–2.1)^†^
API ZYM:		
Esterase (C4)	−	+
Crystine arylamidase	−	w
Naphthol-AS-BI-phosphohydrolase	+	w
N-acetyl-b-glucosaminidase	−	w
Rapid ID 32A:		
Glutamyl glutamic acid arylamidase	+	w
Serine arylamidase	w	+
API 20A:		
Salicin	−	+
D-raffinose	−	+
D-sorbitol	−	w
Major cellular fatty acids (in descending order)	C_18:1_ *ω*9*c*, C_16:0_, C_16:0_ DMA	C_18:1_ *ω*9*c*, C_16:0_, C_18:0_
DNA G + C content (mol%)	62.2	56.9^†^

Strains: 1, *Mesosutterella faecium* AGMB02718^T^; 2, *Mesosutterella multiformis* DSM 106860^T^. All data are from the present study unless otherwise indicated. +, positive; w, weak; and −, negative. ^†^Data were taken from Sakamoto et al. (2018).

### 3.3 Chemotaxonomic features

The cellular fatty acid profiles (>1.0%) of strain AGMB02718^T^ and the reference strain are shown in Table 2. The major fatty acids (>10.0%) of strain AGMB02718^T^ were C_18:1_ *ω*9*c* (24.0%), C_16:0_ (21.4%), and C_16:0_ DMA (11.6%), whereas the major fatty acids of the reference strain *M. multiformis* DSM 106860^T^ were C_18:1_ *ω*9*c* (33.0%), C_16:0_ (20.7%), and C_18:0_ (14.6%). Differences in major and minor fatty acids differentiated strain AGMB02718^T^ from the closely related strain of the genus *Mesosutterella* (Table 2). The respiratory quinones of the strain AGMB02718^T^ were menaquinone 6 (MK-6) and methylmenaquinone 6 (MMK-6), similar to those of the reference strain (Supplementary Table S1).

**Table 2:** Cellular fatty acid compositions of strain AGMB02718^T^ and reference strain grown in TSAB medium.

Fatty acid	1	2
Saturated straight-chain		
C_14:0_	3.08	-
C_16:0_	**21.39**	**20.66**
C_17:0_	-	1.36
C_18:0_	6.63	**14.55**
Dimethyl acetal (DMA)		
C_14:0_	-	-
C_16:0_	**11.58**	3.81
C_18:0_	-	1.06
C_18:1_ *ω*9*c*	8.68	4.34
Aldehyde		
C_16:0_ ALDE	4.08	1.25
Unsaturated straight-chain		
C_18:1_ *ω*9*c*	**23.96**	**32.99**
C_18:2_ *ω*9,12*c*	7.22	5.54
Summed features^*^		
5	3.82	1.06
7	2.91	1.45
10	-	8.12

Major components (>10.0%) are highlighted in bold. Strains: 1, *Mesosutterella faecium* AGMB02718^T^; 2, *Mesosutterella multiformis* DSM 106860^T^. All data were obtained from the current study. Values are percentages of total fatty acids. Fatty acids amounting to <1.0% of the total in all strains are not shown. -, not detected. ^*^Summed features represent groups of fatty acids that could not be separated using the MIDI system. Summed feature compositions: 5, C_15:0_ DMA and/or C_14:0_ 3-OH; 7, C_17:2_ and/or C_17:1_ ω8c; 10, iso-C_18:1_ ω11c/9 t/6 t FAME and/or unknown fatty acid ECL 17.834.

### 3.4 Genome properties and genetic relatedness

**Figure 2:**
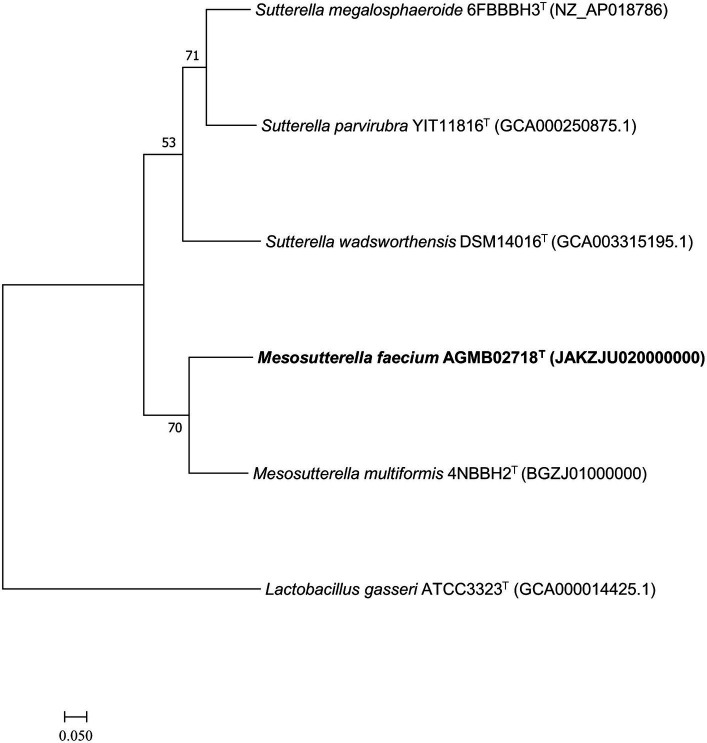
Maximum likelihood (ML) phylogenomic tree based on up-to-date bacterial core gene set (concatenated alignment of 92 core genes) showing the relationship between strain AGMB02718^T^ and closely related taxa. The bootstrap values are indicated at the nodes. Scale bar: 0.050 substitutions per nucleotide position.

Whole-genome sequencing was performed to analyze the genomic characteristics of strain AGMB02718^T^. The assembled genome of the strain AGMB02718^T^ was a single circular chromosome with a coverage of 250.0×. The whole-genome sequence of the novel strain was deposited in NCBI under the accession number JAKZJU020000000 and visualized using the CGView tool (Supplementary Figure S3). Based on the genomic analysis of strain AGMB02718^T^, the genomic GC content was 62.2%, the length of the genome was 2,606,253 bp (N50 value of 2,332,978 bp), which contained 2,278 protein-coding genes and 71 RNA genes, including five 5S rRNA, five 16S rRNA, five 23S rRNA, three non-coding RNA (ncRNA), and 53 tRNA genes (Supplementary Table S3). In addition, 1,801 protein sequences were functionally assigned to categories based on clusters of orthologous group (COG) assignments, revealing that the largest functional category of strain AGMB02718^T^ accounted for function unknown (14.7%), followed by energy production and conversion (10.6%), and amino acid transport and metabolism (9.6%) (Supplementary Table S4). The ANI values between strain AGMB02718^T^ and closely related species including *M. multiformis* 4NBBH2^T^ (GCA_003402575.1), *S. megalosphaeroide* 6FBBBH3^T^ (GCF_003609995.1), *S. wadsworthensis* DSM 14016^T^ (GCA_003315195.1), and *S. parvirubra* YIT 11816^T^ (GCA_000250875.1) were 72.1, 69.1, 67.1, and 68.8, respectively (*S. stercoricanis* CCUG 47620^T^, *P. secunda* YIT 12071^T^, and *P. excrementihominis* YIT 11859^T^ were excluded owing to a lack of whole-genome sequencing information). OrthoANI values based on the entire genome were 72.1–67.1% for the most closely related strains (Supplementary Figure S4). The dDDH relatedness values between strain AGMB02718^T^ and the reference strains were 20.9, 21.5, 20.8, and 19.6% (using Formula 2), respectively. The results for ANI, orthoANI, and dDDH between strain AGMB02718^T^ and closely related strains are listed in Supplementary Table S5. Moreover, a whole-genome-based phylogenomic tree constructed using UBCG (version 3.0) supported that the strain AGMB02718^T^ formed a phylogenetic lineage belonging to the genus *Mesosutterella*, consistent with the 16S rRNA-based phylogenetic tree (Figure 2). Because strain AGMB02718^T^ exhibited significantly low values for the dDDH (<70%) and ANI cutoff (95–96%) proposed for bacterial species delineation (Chun et al., 2018), these genetic analyses collectively classified strain AGMB02718^T^ as a novel strain within the *Mesosutterella* genus.

### 3.5 Genomic features associated with Kdo_2_-lipid A biosynthesis of strain AGMB02718^T^

**Figure 3:**
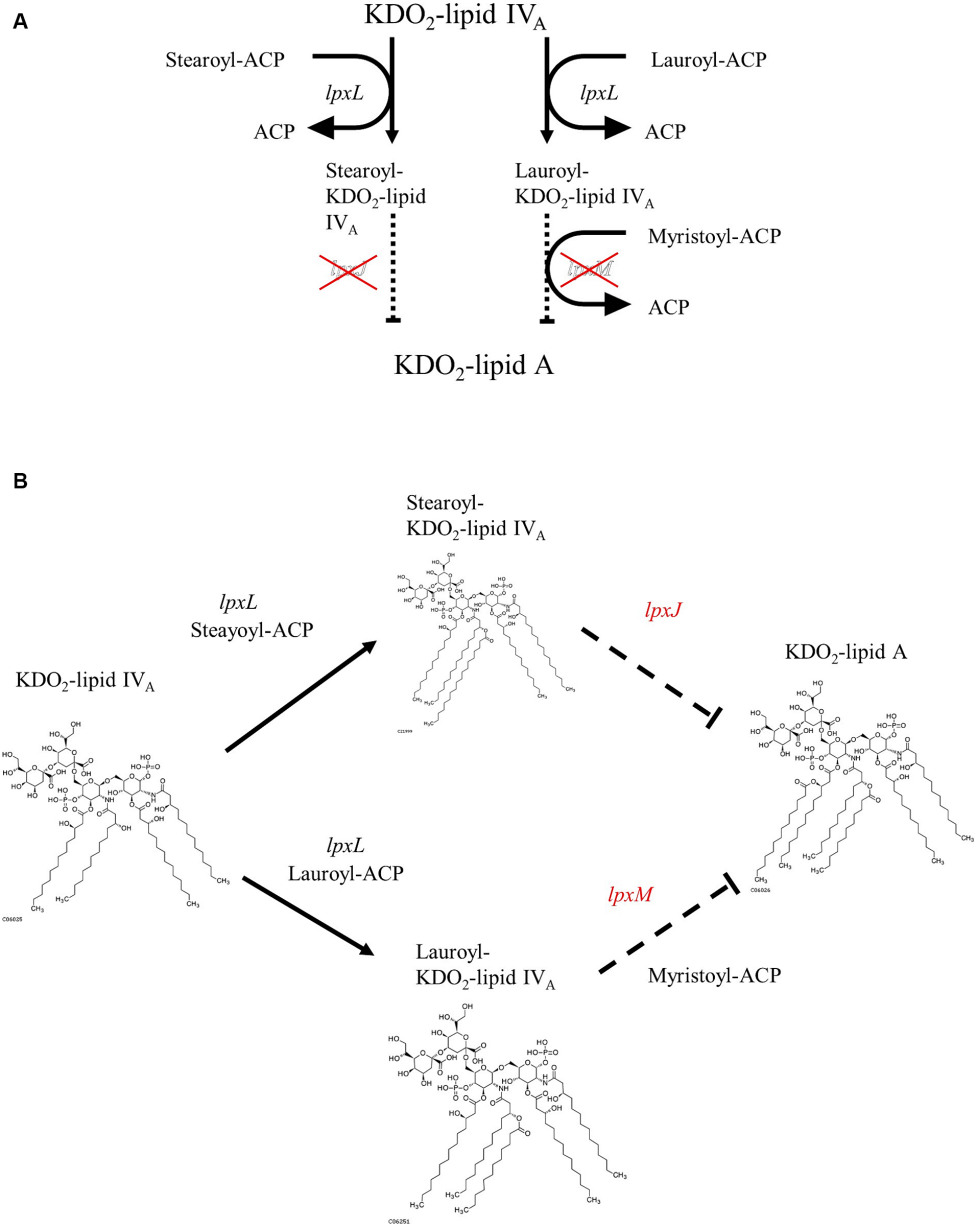
Proposed pathway of Kdo_2_-lipid A biosynthesis from Kdo_2_-lipid IV_A_ in strain AGMB02718^T^. **(A)** Pathway diagram. **(B)** Structure-based schematic diagram.

Differences in LPS structure play an important role in determining its toxicity. Owing to the difference in the structure of lipid A, a component of LPS, *Sutterella* spp. and *E. coli* have different pro-inflammatory properties (Hiippala et al., 2016). Lipid A from *E. coli* is hexa-acylated to activate the TLR4–Mal–MyD88 pathway and is highly toxic, whereas tetra- or penta-acylated lipid A, generated from *Sutterella* spp., signals through TLR4–TRAM-TRIF and is 100-fold less toxic (Park et al., 2009; Herath et al., 2011; Han et al., 2013). Additionally, *E. coli* and related Proteobacteria contain all the nine genes required for complete KLA biosynthesis (Opiyo et al., 2010). *Sutterella* spp.-derived lipid A genomes lack *lpxL* and *lpxM* genes needed for the hexa-acylation of lipids, resulting in penta-acylated or monophosphorylated forms of lipid A (Park et al., 2009; Herath et al., 2011; Han et al., 2013). Because *Mesosutterella faecium* AGMB02718^T^ belongs to the same family as *Sutterellaceae*, we expected that it would have a lipid A biosynthetic pathway similar to that of the genus *Sutterella*. Therefore, the sequenced genome of the strain AGMB02718^T^ was examined for LPS biosynthesis. Whole-genome mining of the strain AGMB02718^T^ was performed using BlastKOALA, and the following sections were predicted from the genome sequences. The genes associated with LPS biosynthesis, including *lpxA* (UDP-N-acetyl glucosamine acyltransferase [EC:2.3.1.129]), *lpxC* (UDP-3-O-acyl-N-acetylglucosamine deacetylase [EC:3.5.1.108]), *lpxD* (UDP-3-O-acyl-glucosamine N-acyltransferase [EC:2.3.1.191]), *lpxH* (UDP-2,3-diacylglucosamine hydrolase [EC:3.6.1.54]), *lpxB* (lipid-A-disaccharide synthase [EC:2.4.1.182]), *lpxK* (tetraacyldisaccharide 4′-kinase [EC:2.7.1.130]), *waaA* (lipid IV_A_ 3-deoxy-D-manno-octulosonic acid transferase [EC 2.4.99.12] or (Kdo)-lipid IV_A_ 3-deoxy-D-manno-octulosonic acid transferase [EC 2.4.99.13]), and *lpxL* (Kdo_2_-lipid IV_A_ lauroyltransferase/acyltransferase [EC 2.3.1.241]) were detected; however, the *lpxJ* and *lpxM* genes, responsible for synthesizing KLA from lauroyl or stearoyl-Kdo_2_-lipid IV_A_, were absent (Supplementary Table S6). These results suggest that strain AGMB02718^T^ is unable to biosynthesize hexa-acylated lipid A (Figure 3).

### 3.6 *Mesosutterella faecium* AGMB02718^T^ induces a low-level inflammatory cytokine response in macrophages

**Figure 4:**
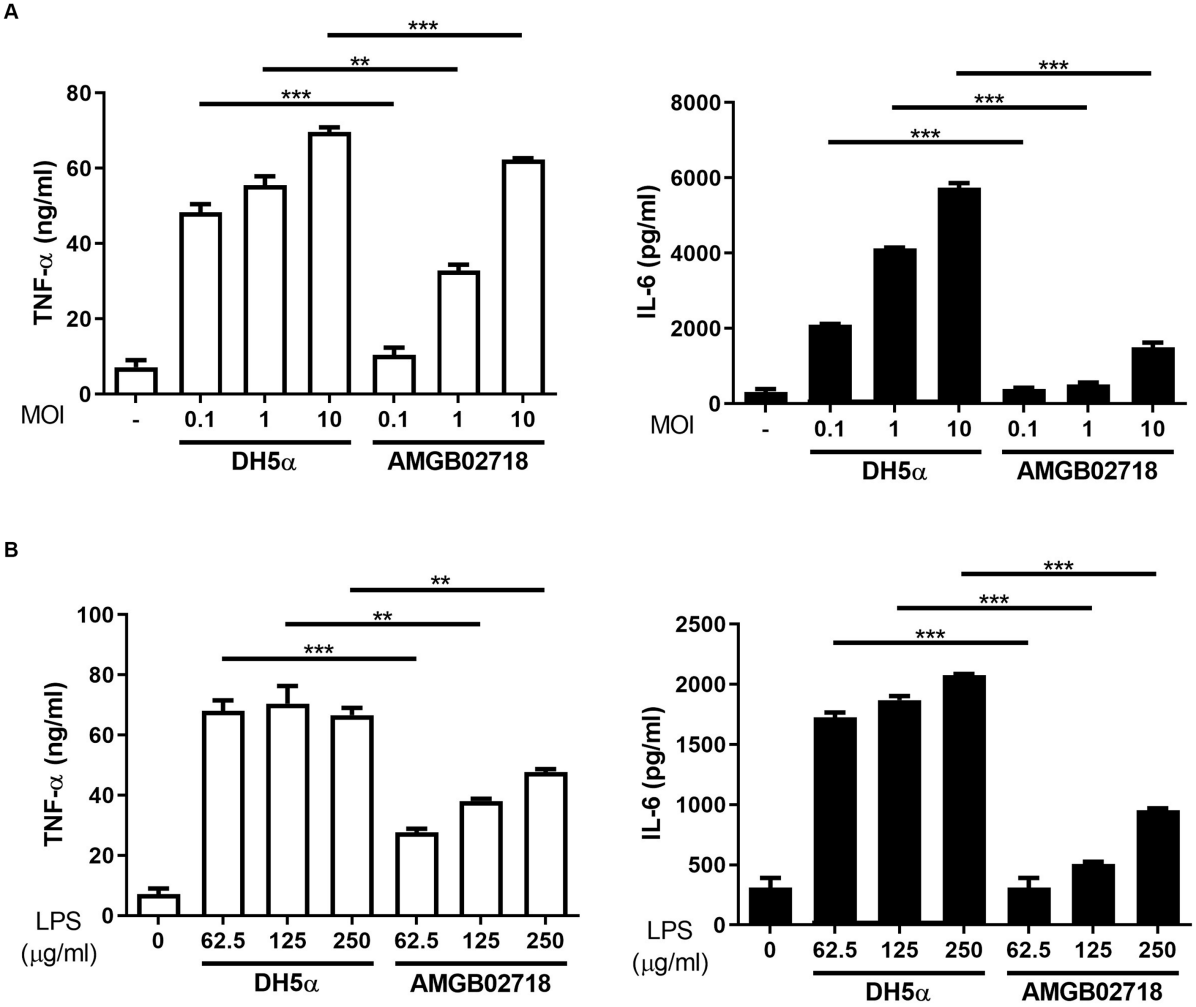
Inflammatory cytokine production in RAW264.7 cells induced by bacterial treatment. RAW264.7 cells were treated with **(A)** *Escherichia coli* and AGMB02718^T^ at MOIs of 0.1, 1, and 10 or **(B)** LPS at 0, 62.5, 125, and 250 μg/mL for 6 h. Cell supernatants were then analyzed for IL-6 and TNF-α using ELISA. All data are presented as means ± SEM. Statistically significant values were calculated by comparison with the *E. coli* group. ^**^p < 0.01; ^***^p < 0.001 by Student’s *t*-test.

The administration of mouse macrophages with *E. coli* (hexa-acylated lipid A) induced higher expression of inflammatory cytokines than *Bordetella pertussis* (penta-acylated lipid A), owing to the different forms of lipid A (Ernst et al., 2021). From the results of the whole-genome analysis, it can be expected that the strain AGMB02718^T^ produces LPS with low toxicity. To evaluate whether the strain AGMB02718^T^ has low toxicity, its inflammatory stimulatory activity was first examined using live bacteria. The result showed that the *E. coli*-treated group significantly induced pro-inflammatory cytokines (IL-6 and TNF-α) at all concentrations of MOIs, whereas the strain AGMB02718^T^-treated group did not stimulate macrophages at a similar level as the non-treated group at all MOIs (Figure 4A). In addition, the inflammatory stimulation activity was examined using LPS isolated from the bacteria, and it was determined that the LPS of strain AGMB02718^T^ was less toxic than that of *E. coli* (Figure 4B).

### 3.7 *Mesosutterella faecium* AGMB02718^T^ prevents DSS-induced colitis

**Figure 5:**
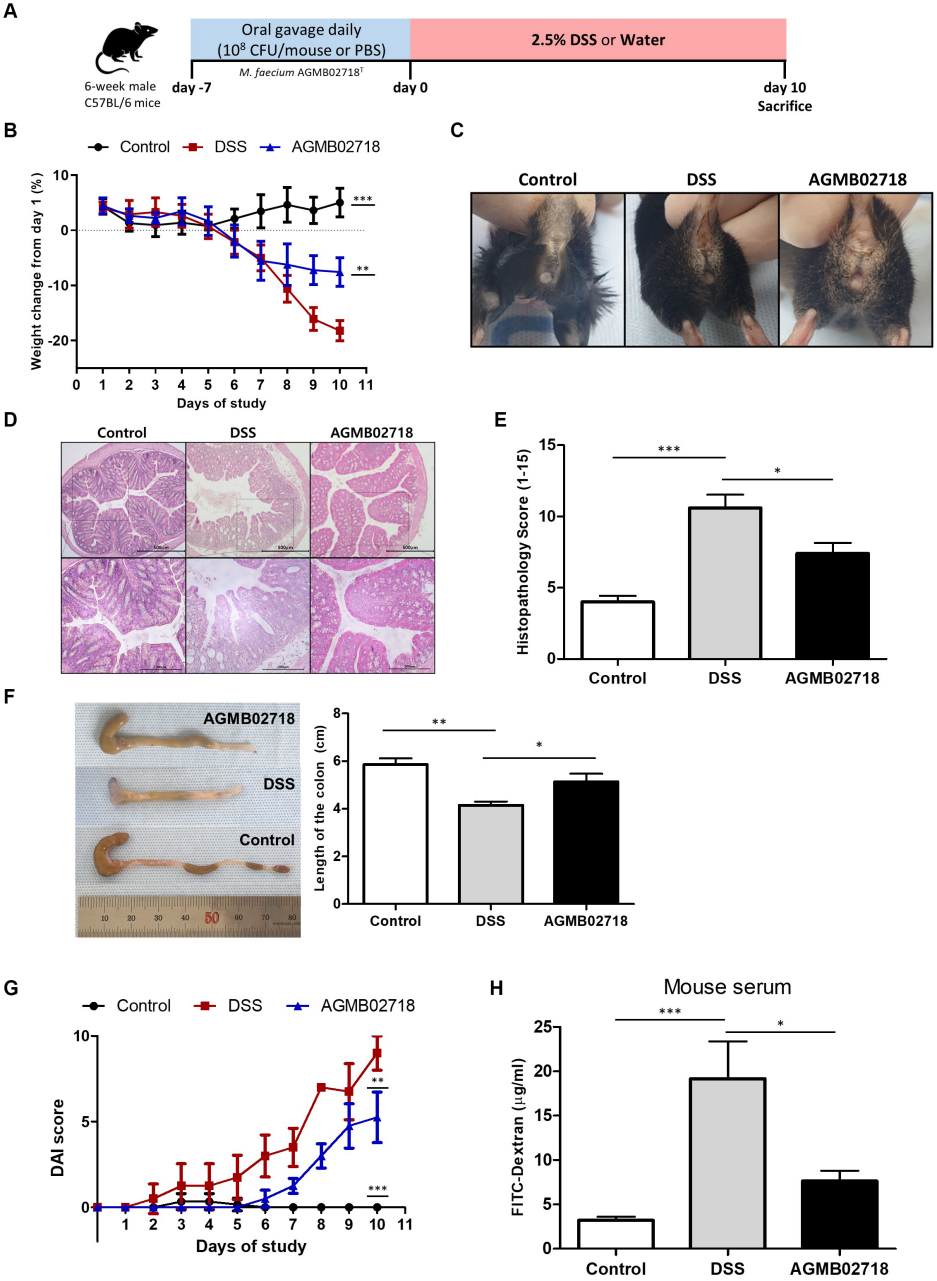
Strain AGMB02718^T^ ameliorates DSS-induced colitis. **(A)** Schematic diagram of the DSS-induced colitis mouse model. **(B)** Percentage of mouse weight change from days 0 to 10. **(C)** Representative pictures showing bloody stools. **(D)** H&E staining of control and colitis mice **(E)** and the histopathology scores (0–15) in the three groups. **(F)** Colon lengths of the tree groups on day 10. **(G)** Disease activity index (DAI) values calculated with time represent the DAI scores among the groups at day 10. **(H)** Bar chart showing the plasma concentration of FITC-dextran among three groups. All data are presented as means ± SEM. Statistically significant values were calculated by comparison with the DSS group. ^*^p < 0.05; ^**^p < 0.01; ^***^p < 0.001 by One-way ANOVA with Tukey’s multiple comparisons.

A non-excessive inflammatory response by intestinal mucosal microbes is important to induce an optimal host immune response (Pandiyan et al., 2019). This is related to the characteristics of the family *Sutterellaceae*, including the genera *Sutterella*, *Mesosutterella*, and *Parasutterella*, which are commensal bacteria that adhere to the intestine (Hiippala et al., 2016). Tetra- or penta-acylated lipid A plays an important role in the host immune response by not exceeding the inflammatory response threshold owing to its attenuated toxicity (Di Lorenzo et al., 2020). This claim is supported by earlier findings showing that mice lacking immune regulation develop more severe DSS-induced colitis (Keubler et al., 2015). The results of the LPS toxicity test confirmed that AGMB02718^T^-derived LPS treatment induced low levels of inflammation *in vitro*. Taken together, these studies suggest that the strain AGMB02718^T^ possessing all these characteristics may have ideal effects against IBD. To this end, we established a mouse model of DSS-induced colitis to test the *in vivo* activity of the AGMB02718^T^ strain. After pretreatment with bacteria or PBS, IBD was induced using DSS for 10 days (Figure 5A). Although the body weights of the mice in the DSS group were consistently reduced from day 2, the mice in the AGMB02718 group showed a significant improvement in weight loss, although there was an apparent decrease compared to the control group (non-DSS-induced). At the end of the experiment, the weight in the DSS, AGMB02718, and control groups was decreased by −18.21 ± 1.85%, −7.58 ± 2.61%, and increased by +5.03 ± 2.60%, respectively (Figure 5B). The DSS group had bloody stools with diarrhea, whereas mild diarrhea was observed in the bacterial treatment group; however, bloody stools were prevented (Figure 5C). Treatment with strain AGMB02718^T^ significantly reduced colonic architecture disruption compared with DSS treatment (Figure 5D). Consistent with these results, the histopathology scores of the AGMB02718 group were significantly lower than those of the DSS-treated group (7.40 ± 1.50 vs. 10.60 ± 1.85) (Figure 5E). A common feature of the DSS-induced colitis model is a decrease in colon length and an increase in the DAI. Although the DSS treatment decreased the colon length of the mice (4.15 ± 0.31 cm) compared to the control group (5.86 ± 0.52 cm), the strain AGMB02718^T^ treatment reversed this effect (5.14 ± 0.66 cm) (Figure 5F). The DAI values were evaluated based on the physiological signs (weight loss, diarrhea, and occult/gross bleeding) induced by 2.5% DSS treatment for 10 days, showing that increased DAI scores in the DSS group (9.00 ± 1.00) were markedly suppressed in the AGMB02718 group (5.25 ± 1.48) (Figure 5G). In addition, intestinal permeability was investigated using FITC-dextran analysis. As a result, the permeability was decreased in the mice of the AGMB02718 group (5.55 ± 1.48 μg/mL) compared to the DSS group (19.18 ± 7.29 μg/mL), suggesting that the intestinal barrier function was improved after treatment with strain AGMB02718^T^ (Figure 5H). Collectively, it was confirmed that administration of *M. faecium* AGMB02718^T^ prevented body weight and colon length reduction and protected and maintained overall intestinal integrity, suggesting that strain AGMB02718^T^ can mitigate DSS-induced colitis symptoms. However, there are still several limitations to conclusively stating that this preventive effect is entirely due to low-toxicity LPS. To overcome this limitation, further studies are needed, such as the direct administration of LPS derived from *M. faecium* AGMB02718^T^ at an *in vivo* level, investigation of various immune cells including macrophages in the colon, and investigation of inflammation levels in the colon. Nevertheless, there still exists the possibility of alternative mechanisms for IBD prevention through the administration of *M. faecium* AGMB02718^T^.

### 3.8 *Mesosutterella faecium* AGMB02718^T^ alleviates DSS-induced microbiome dysbiosis and reconstitutes the gut bacteria community

**Figure 6:**
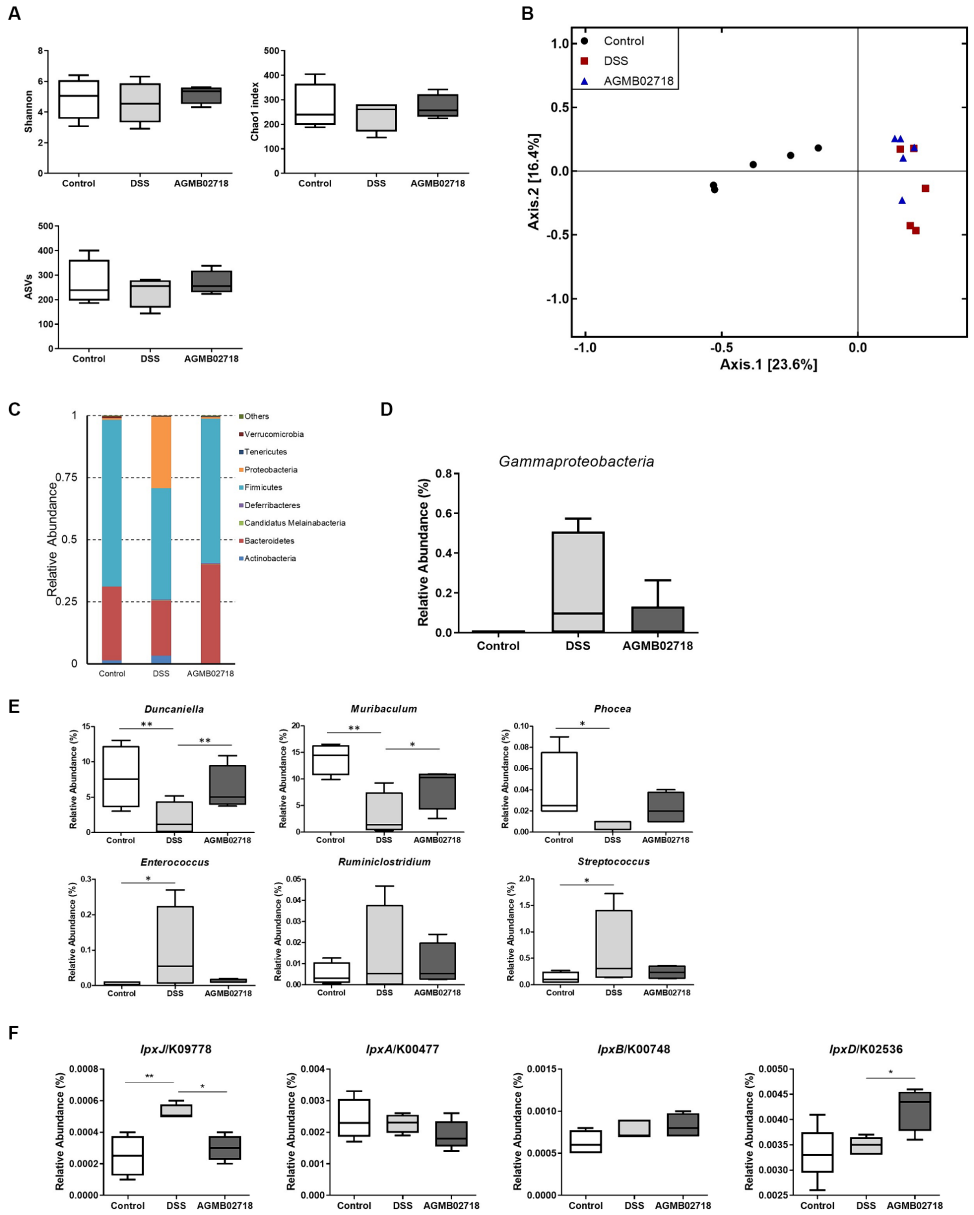
Strain AGMB02718^T^ prevents DSS-induced gut microbiota dysbiosis (*n* = 5/group). **(A)** Alpha diversity index (ASVs, Chao1, and Shannon) among the three groups. **(B)** 2D PCoA analysis among the three groups based on Jaccard index method. Each plot represents one sample. **(C)** Component proportion of the gut microbiota at the phylum level. **(D)** Class Gammaproteobacteria ratio among three groups. **(E)** Relative abundance of IBD-positive (*Duncaniella*, *Muribaculum*, and *Phocea*) and negative (*Enterococcus*, *Ruminiclostridium*, and *Streptococcus*) genera among three groups. **(F)** Comparison of the abundance of LPS-synthesis genes among three groups through tax4fun analysis. Statistically significant values were calculated by comparison with the DSS group. ^*^p < 0.05; ^**^p < 0.01 by One-way ANOVA with Tukey’s multiple comparisons.

To further investigate the protective effect of strain AGMB02718^T^ administration in a DSS-induced mouse model, we evaluated changes in the gut microbiota in mouse feces. Fecal samples were collected from each group (*n* = 5) after DSS treatment (day 10). A total of 452,589 clean reads (control = 142,265; DSS = 160,271; and AGMB02718 = 150,053) were obtained, and 3,071 identified ASVs (control = 1,064; DSS = 935; and AGMB02718 = 1,072) were clustered. For microbiota diversity analysis, alpha-diversity between groups was compared and analyzed using ASVs, Chao1, and Shannon. Alpha diversity is decreased in patients with CD or UC (Sokol and Seksik, 2010). The fecal microbiota of mice administered only DSS (DSS group) showed reduced alpha-diversity compared to that of the control group, as previously reported; however, interestingly, the reduced alpha-diversity was slightly recovered in the group administered the strain AGMB02718^T^ (Figure 6A). Next, a PCoA analysis based on the Jaccard index was performed to compare beta diversity in the gut microbiota. As shown in Figure 6B, ANOSIM analysis indicated that the Control group was separated from the other two groups, and the AGMB02718 group and DSS group were marginally changed (*R*-value = 0.60889, *p* value = Not significant).

It has been known that the relative abundance of bacteria taxa was altered in individuals with IBD and in DSS-induced mouse (Zuo and Ng, 2018). The abundance of taxa at the phylum, class, family, and genus levels differed between groups (Figure 6C; Supplementary Figure S5). Specifically, at the phylum level, the abundance of Proteobacteria increased in the DSS group compared to the control group as in a previous study, whereas this dysbiosis was reversed by AGMB02718^T^ administration (Figure 6C). In particular, the abundance of Gammaproteobacteria increased in the DSS group (22.32%) compared to the control group (0.06%), but the abundance of these bacteria reduced after AGMB02718^T^ treatment (5.36%) (Figure 6D). To further support the effect of strain AGMB02718^T^ on maintaining intestinal microbial homeostasis, we continued to explore the specific bacterial genera showing stable changes during IBD, in which the abundances of some bacterial genera including *Duncaniella*, *Muribaculum*, and *Phocea* decrease, but the abundances of *Enterococcus*, *Ruminiclostridium*, and *Streptococcus* increase compared with those of a healthy model (Teitelbaum and Triantafyllopoulou, 2006; Bian et al., 2019; Dobranowski et al., 2019; Zhou et al., 2020; Forster et al., 2022; Pisani et al., 2022; Thomas et al., 2022). This imbalance in the gut microbiota induced by IBD occurred in DSS-induced mice; however, AGMB02718^T^ administration alleviated DSS-induced dysbiosis (Figure 6E). Additionally, functional profiling of the gut microbial communities in each group was predicted based on 16S rRNA sequencing data using tax4fun analysis. Predictions of LPS synthesis genes for each group were conducted through KEGG pathway analysis. As a result, the gene *lpxJ*, associated with toxicity in LPS, was significantly enriched in the DSS group compared to the other two groups. Conversely, excluding *lpxD* among the non-toxicity-related genes, there was almost no difference in *lpxA* and *lpxB* between the three groups (Figure 6F). Meanwhile, according to reports, gut dysbiosis can lead to gut leakage, damaging the epithelial barrier and allowing gut contents to come into contact with the host, thus inducing an inflammatory response (Walker and Lawley, 2013). These research findings underscore the importance of maintaining gut integrity in suppressing additional inflammatory reactions. Furthermore, based on existing reports, due to the characteristic of the family *Sutterellaceae* adhering to the mucosa, more strains of the *Sutterella* genus are found in mucosa covered with mucus than in those without (Tajima et al., 2022). In summary, the destruction of the gut structure induces the detachment of *Sutterella* from the gut mucosa and triggers colonic inflammation. These facts provide a comprehensive explanation for our findings of increased colon permeability (Figure 5H) and the presence of *Sutterellaceae* in the feces of the DSS group (Supplementary Figure S6). Because we did not investigate the gut mucosal microbiota, we cannot definitively claim whether *M. faecium* AGMB02718^T^ has colonized in the gut. However, considering the significant decrease in gut permeability in the AGMB02718 group, and the almost non-detection of *Sutterellaceae* in fecal metagenome analysis, we can indirectly infer the possibility of *M. faecium* AGMB02718^T^ colonized in the gut.

Taken together, the gut microbiota analysis suggests that the administration of strain AGMB02718^T^ may prevent DSS-induced colitis by regulating the gut bacterial community.

### 3.9 Conclusion

In this study, strain AGMB02718^T^ was isolated from swine feces and identified as a novel member of the genus *Mesosutterella* based on phenotypic, physiological, phylogenetic, and biochemical analyses. Interestingly, whole-genome sequencing analysis revealed that the genome of the isolate does not include genes responsible for the high toxicity of LPS, suggesting that strain AGMB02718^T^ may produce low-toxicity LPS and consequently help maintain host immune homeostasis, as previously mentioned. Thus, we investigated this hypothesis using a DSS-induced colitis and found that strain AGMB02718^T^ can protect the colitis induced by DSS. In addition, the isolate contributed to the maintenance of host immune balance and intestinal microbial community homeostasis. This study identified a new member of the genus *Mesosutterella* that may be used as a next-generation probiotic to control IBD.

### 3.10 Description of *Mesosutterella faecium* sp. nov

*Mesosutterella faecium* sp. nov. (fae’ci.um. L. fem. Gen. pl. n. *faecium*, derived from feces).

Colonies grown on TSAB plate are white, circular (0.5–1 mm in diameter), smooth, and convex after a 72 h culture at 37°C. Cells (0.4 μm in width and 1.0–1.5 μm in length) are coccobacilli-shaped, strictly anaerobic, Gram stain-negative, non-motile, flagella-lacking, catalase- and oxidase-negative. Growth occurs at 25–45°C (optimal 37°C) and with 0.5–1% NaCl (optimal 0.5%). Strain AGMB02718^T^ is positive for the following enzyme activities in the API ZYM and Rapid ID 32A strips: mannose fermentation, raffinose fermentation, nitrate reduction, alkaline phosphatase, arginine arylamidase, proline arylamidase, leucyl glycine arylamidase, phenylalanine arylamidase, leucine arylamidase, pyroglutamic acid arylamidase, tyrosine arylamidase, alanine arylamidase, glycine arylamidase, histidine arylamidase, glutamyl glutamic acid arylamidase, and serine arylamidase. However, it is negative for urease, arginine dihydrolase, α-galactosidase, β-galactosidase, β-galactosidase 6-phosphate, α-glucosidase, β-glucosidase, α-arabinosidase, β-glucuronidase, β-N-acetyl-glucosaminidase, glutamic acid decarboxylase, α-fucosidase, and indole production. In the API 20A strips, all tests are negative. The major fatty acids detected in AGMB02718^T^ are C_18:1_*ω*9*c*, C_16:0_, and C_16:0_ DMA. Respiratory quinones are MK-6 and MMK-6.

GenBank accession numbers for the 16S rRNA gene and whole-genome sequences of strain AGBM02718^T^ are OM971902 and JAKZJU020000000, respectively. The strain was deposited in the Korea Collection for Type Culture (KCTC 25541^T^) and Guangdong Microbial Culture Collection Center (GDMCC 1.2717^T^).

## Data availability statement

The datasets presented in this study can be found in online repositories. The names of the repository/repositories and accession number(s) can be found at: https://www.ncbi.nlm.nih.gov/genbank/, OM971902 and JAKZJU020000000.

## Ethics statement

The animal study was approved by Institutional Animal Care and Use Committee of the Korea Research Institute of Bioscience and Biotechnology (approval number: KRIBB-AEC-22146). The study was conducted in accordance with the local legislation and institutional requirements.

## Author contributions

SY: Conceptualization, Data curation, Investigation, Methodology, Software, Writing – original draft. BO: Investigation, Methodology, Writing – review & editing. SR: Investigation, Methodology, Writing – review & editing. JB: Investigation, Methodology, Writing – review & editing. EH: Investigation, Methodology, Writing – review & editing. JM: Investigation, Methodology, Writing – review & editing. J-HJ: Investigation, Methodology, Writing – review & editing, Conceptualization. JL: Funding acquisition, Investigation, Methodology, Resources, Writing – review & editing, Conceptualization.

## Footnotes

^1^ https://www.ezbiocloud.net/

^2^ https://cgview.ca/
