# Supplementary material for: Novel microbiota Mesosutterella faecium sp. nov. has a protective effect against inflammatory bowel disease
Source: Front Microbiol. 2024 Apr 2;15:1342098. doi: 10.3389/fmicb.2024.1342098 (PMC11022602; doi:10.3389/fmicb.2024.1342098)
Supplement: Supplementary file 1 [file Data_Sheet_1.pdf]

Frontiers in Microbiology

Novel microbiota *Mesosutterella faecium* sp. nov. has a protective effect against inflammatory bowel disease

Seung Yeob Yu[1,2], Byeong Seob Oh[1], Seoung Woo Ryu[1,2], Jeong Eun Bak[1,2], Eun Seo Heo[1,3], Jeong Chan Moon[4], Jae-Ho Jeong[2*], and Ju Huck Lee[1,3*]

[1]Korean Collection for Type Cultures, Biological Resource Center, Korea Research Institute of Bioscience and Biotechnology, Jeongeup, Republic of Korea

[2]BioMedical Sciences Graduate Program (BMSGP), Chonnam National University, Hwasun, Republic of Korea

[3]University of Science and Technology (UST), Daejeon, Republic of Korea

[4]National Institute of Ecology, Yeongyang, Republic of Korea

Running header: *Mesosutterella faecium* sp. nov. protects DSS-induced colitis

Keywords: gut microbiota, *Mesosutterella*, inflammation, inflammatory bowel disease, murine colitis model

***Corresponding authors**

Jae-Ho Jeong, Ph.D. Tel: +82-61-379-2747, Fax: +82-62-232-9708, E-mail: jeongjaeho@jnu.ac.kr

Ju Huck Lee, Ph.D. Tel: +82-63-570-5634, Fax: +82-63-570-5609**,** E-mail: juhuck@kribb.re.kr

Supplementary Materials
Supplementary Tables

Biochemical analyses	AGMB02718^T
Growth temperature (℃)	
15	-
20	-
25	+
30	++
37	+++
40	++
45	+
50	-
Salt tolerance test (NaCl, %)	
0.5	+++
1	++
2	-
3	-
4	-
5	-
Catalase :	-
Oxidase :	-
Respiratory quinones :	MK-6, MMK-6

Supplementary Table S1. Biochemical characterization of strain AGMB02718^T. Growth temperature, salt tolerance, catalase, oxidase, and respiratory quinone analyses. Cells were grown in TSAB for 3 days at 37 ℃ in anaerobic conditions. +++, strongly positive; ++, positive; +, weakly positive; −, negative.

Characteristics	1	2
Acid production (API 20A) from :		
L-tryptophane	-	-
urea	-	-
D-glucose	-	-
D-mannitol	-	-
D-lactose (bovine origin)	-	-
D-saccharose (sucrose)	-	-
D-maltose	-	-
salicin	-	+
D-xylose	-	-
L-arabinose	-	-
gelatin (bovine origin)	-	-
esculin<br>ferric citrate	-	-
glycerol	-	-
D-cellobiose	-	-
D-mannose	-	-
D-melezitose	-	-
D-raffinose	-	+
D-sorbitol	-	w
L-rhamnose	-	-
D-trehalose	-	-

Characteristics	1	2
Enzymatic activity (Rapid ID 32A and ZYM)		
Urease	-	-
Arginine dihydrolase	-	-
α-Galactosidase	-	-
β-Galactosidase	-	-
β-Galactosidase 6-phosphate	-	-
α-Glucosidase	-	-
β-Glucosidase	-	-
α-Arabinosidase	-	-
β-Glucuronidase	-	-
β-N-acetyl-glucosaminidase	-	-
Mannose fermentation	+	+
Raffinose fermentation	+	+
Glutamic acid decarboxylase	-	-
α-Fucosidase	-	-
Nitrate reduction	+	+
Indole production	-	-
Alkaline phosphatase	+	+
Arginine arylamidase	+	+
Proline arylamidase	w	w
Leucyl glycine arylamidase	w	w
Phenylalanine arylamidase	+	+
Leucine arylamidase	+	+
Pyroglutamic acid arylamidase	w	w
Tyrosine arylamidase	+	+
Alanine arylamidase	+	+
Glycine arylamidase	+	+
Histidine arylamidase	+	+
Glutamyl glutamic acid arylamidase	+	w
Serine arylamidase	w	+
Esterase (C4)	-	+
Esterase lipase (C8)	w	w
Lipase (C14)	-	-
Valine arylamidase	-	-
Crystine arylamidase	-	w
Trypsin	-	-
α-chymotrypsin	-	-
Acid phosphatase	+	+
Naphthol-AS-BI-phosphohydrolase	+	w
α-mannosidase	-	-

Supplementary Table S2. API 20A, Rapid ID 32A, and ZYM for AGMB02718^T and the reference strain. Strains: 1, AGMB02718^T; 2, *M. multiformis* DSM 106860^T. All data were obtained in this study, and for duplicates, only one experimental result is listed. Cells were grown in TSAB for 3 days at 37 °C in anaerobic conditions. +, positive; w, weak; −, negative.

Properties	Values
**Genome assembly**	
Assemble method	Flye 2.9.2, SPAdes 3.13.1
Sequencing technology	PacBio Sequel, Illumina NovaSeq
Annotation	NCBI PGAP
**Genome features**	
Genome size (bp)	2,606,253
Genome coverage	250.0×
G+C content (%)	62.2
No. of contigs	3
rRNA genes	15
tRNA genes	53
Open reading frame	2,278
CDS assigned by COG	1,801
GenBank Accession No.	JAKZJU020000000

Supplementary Table S3. Genomic characteristics of *Mesosutterella faecium* strain AGMB02718^{T}.

COG	Description	Number of Genes	Percentage (%)
J	Translation, ribosomal structure and biogenesis	149	8.27
A	RNA processing and modification	1	0.06
K	Transcription	132	7.33
**L**	**Replication, recombination and repair**	**180**	**9.99**
N	Cell motility	1	0.06
D	Cell cycle control, cell division, chromosome partitioning	31	1.72
V	Defense mechanisms	25	1.39
T	Signal transduction mechanisms	41	2.28
M	Cell wall/membrane/envelope biogenesis	121	6.72
U	Intracellular trafficking, secretion, and vesicular transport	56	3.11
O	Posttranslational modification, protein turnover, chaperones	66	3.66
**C**	**Energy production and conversion**	**190**	**10.55**
G	Carbohydrate transport and metabolism	40	2.22
**E**	**Amino acid transport and metabolism**	**173**	**9.61**
F	Nucleotide transport and metabolism	79	4.39
H	Coenzyme transport and metabolism	101	5.61
I	Lipid transport and metabolism	46	2.55
P	Inorganic ion transport and metabolism	92	5.11
Q	Secondary metabolites biosynthesis, transport and catabolism	13	0.72
S	Function unknown	264	14.66
**Total**		**1801**	**100**

Supplementary Table S4. Clusters of orthologous group (COGs) functional category analysis of 1,801 genes. The three major parts of the genes were COG categories C (energy production and conversion), E (amino acid transport and metabolism), and L (replication, recombination, and repair).

Assembly No.	Strain	ANI	orthoANI	dDDH
GCA_003402575.1	*Mesosutterella multiformis* 4NBBH2^{T}	72.88	72.13	20.9
GCF_003609995.1	*Sutterella megalosphaeroide* 6FBBBH3^{T}	70.30	69.10	21.5
GCA_003315195.1	*Sutterella wadsworthensis* DSM 14016^{T}	68.53	68.78	20.8
GCA_000250875.1	*Sutterella parvirubra* YIT 11816^{T}	70.40	67.07	19.6

Supplementary Table S5. ANI, orthoANI (%), and dDDH (%) values between AGMB02718^{T} and closely related type strains. Data were calculated using ANI calculator, Orthologous Average Nucleotide Identity Tool (OAT) software, and the GGDC web server.

Gene name	Description	EC number	Locus tag	AGMB02718^T
LpxA	UDP-N-acetylglucosamine acyltransferase	EC:2.3.1.129	MUN46_RS06795	+
LpxC	UDP-3-O-acyl-N-acetylglucosamine deacetylase	EC:3.5.1.108	MUN46_RS01520	+
LpxD	UDP-3-O-acyl-glucosamine N-acyltransferase	EC:2.3.1.191	MUN46_RS06785	+
LpxH	UDP-2,3-diacylglucosamine hydrolase	EC:3.6.1.54	MUN46_RS05595	+
LpxB	lipid-A-disaccharide synthase	EC:2.4.1.182	MUN46_RS06800	+
LpxK	tetraacyldisaccharide 4'-kinase	EC:2.7.1.130	MUN46_RS00875	+
WaaA	lipid IV_A 3-deoxy-D-manno-octulosonic acid transferase	EC 2.4.99.12	MUN46_RS03340	+
	(Kdo)-lipid IV_A 3-deoxy-D-manno-octulosonic acid transferase	EC 2.4.99.13		+
LpxL	Kdo_2-lipid IV_A lauroyltransferase/acyltransferase	EC 2.3.1.241	MUN46_RS02870	+
LpxM	lauroyl-Kdo_2-lipid IV_A myristoyltransferase	EC:2.3.1.243	−	−
LpxJ	Kdo_2-lipid IV_A 3' secondary acyltransferase	EC:2.3.1.-	−	−

Supplementary Table S6. Genes encoding known Kdo_2-lipid A biosynthesis enzymes predicted in the AGMB02718^T genome. +, positive; −, negative.

Supplementary Figure

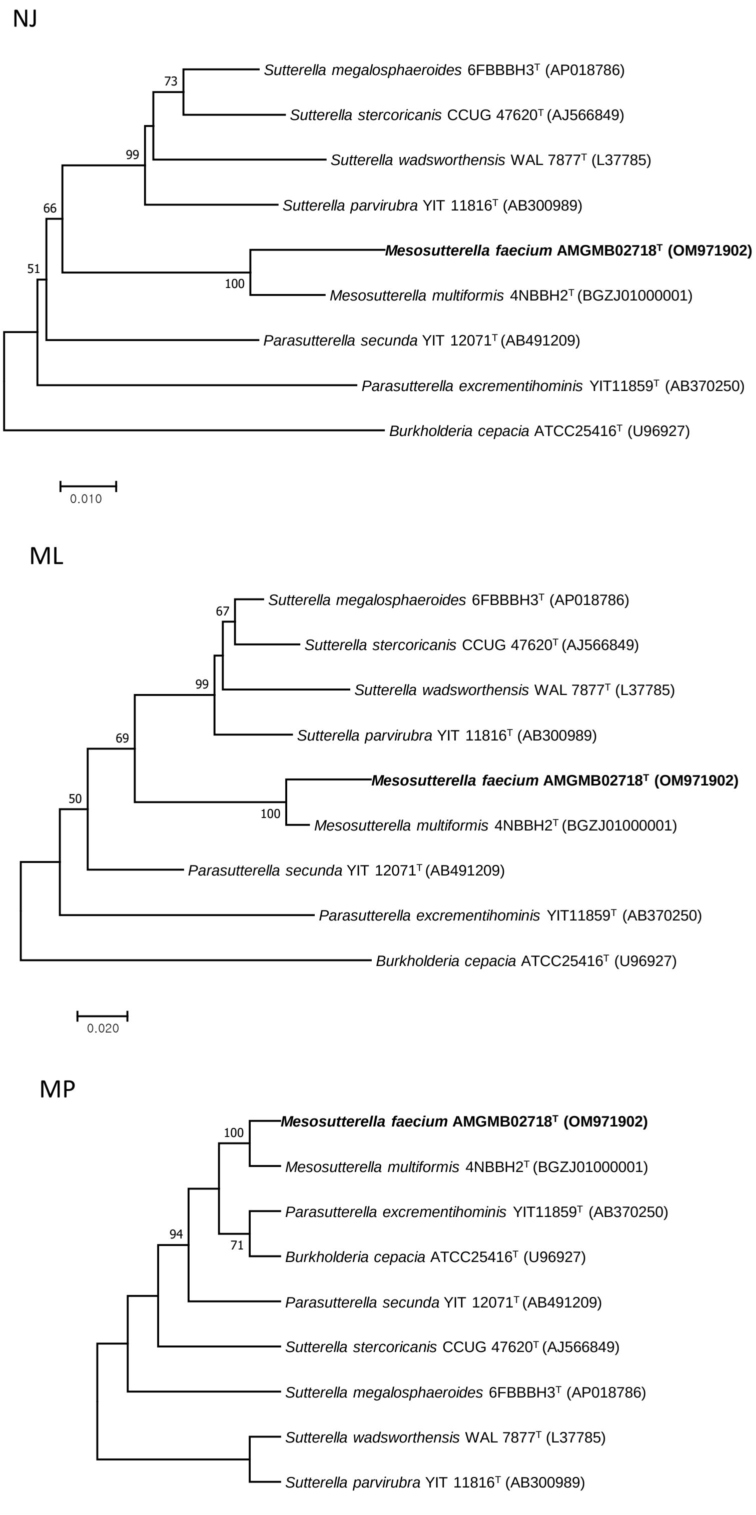

Supplementary Figure S1. Phylogenetic trees based on 16S rRNA gene sequences showing the position of strain AGMB02718^{T}. Bootstrap values (>50%) were calculated using the neighbor-joining (NJ), maximum likelihood (ML), and minimum parsimony (MP) algorithms. Scale bar: 0.010 (NJ), 0.020 (ML) substitutions per nucleotide position.

Supplementary Figures

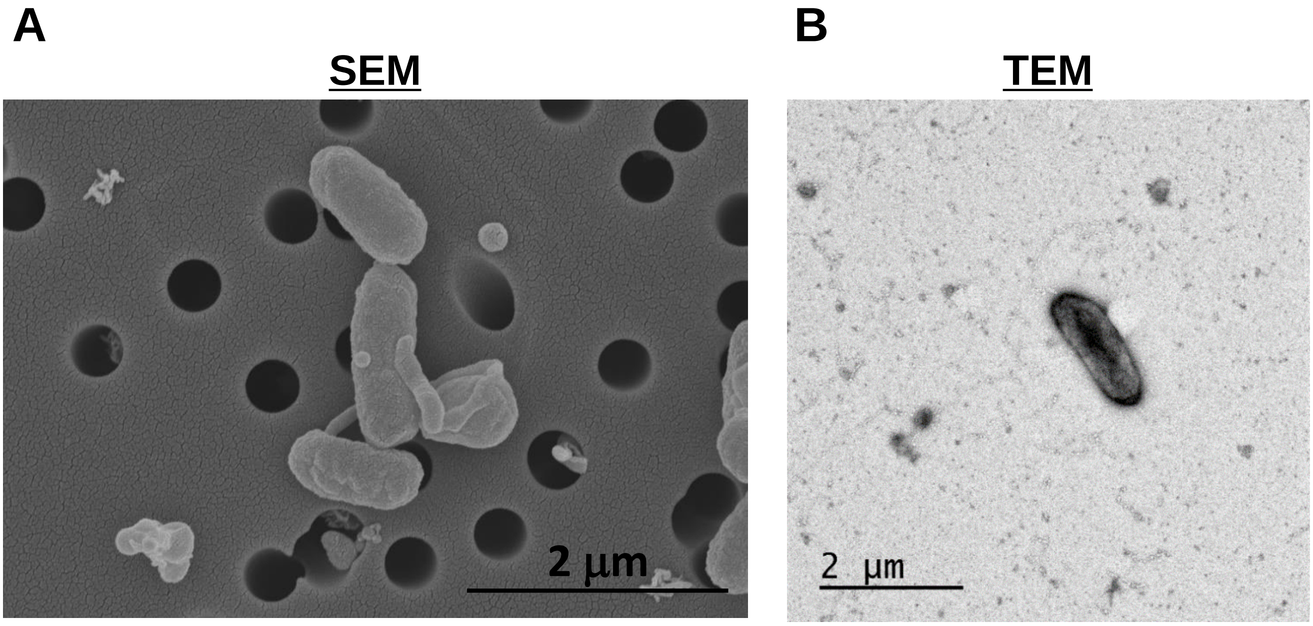

Supplementary Figure S2. Cell morphology of strain AGMB02718^T. Cells were grown in TSAB plates at 37 ℃ for 3 days in anaerobic conditions. Cells were analyzed using **(A)** scanning electron microscopy and **(B)** transmission electron microscopy. Bar indicates 2 μm.

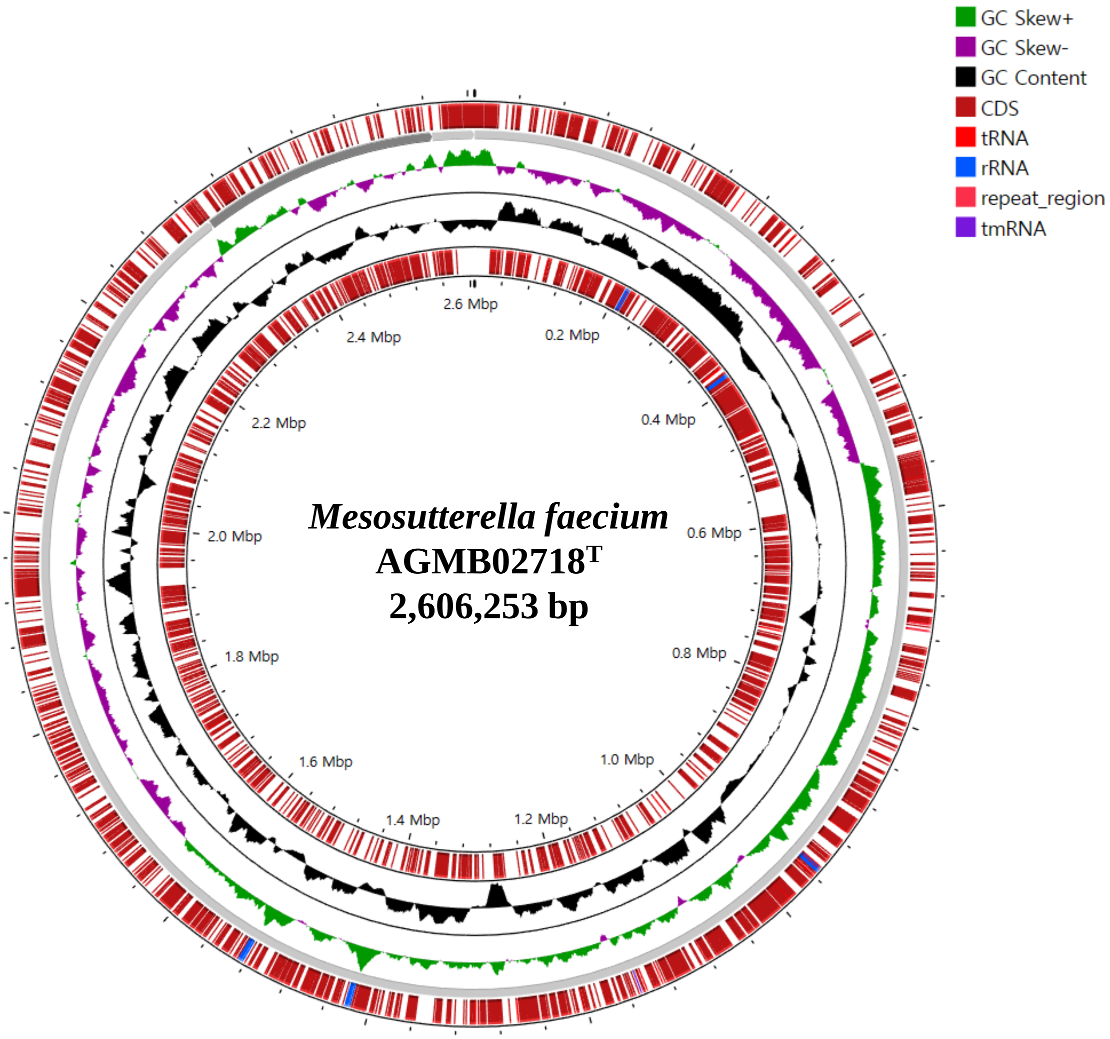

Supplementary Figure S3. Map of the AGMB02718^T genome generated with CGView. From outside to the center: region coding genes (wine), GC skew (green/mauve), GC content (black), tRNA genes (red), rRNA genes (blue), and tmRNA genes (lightmauve).

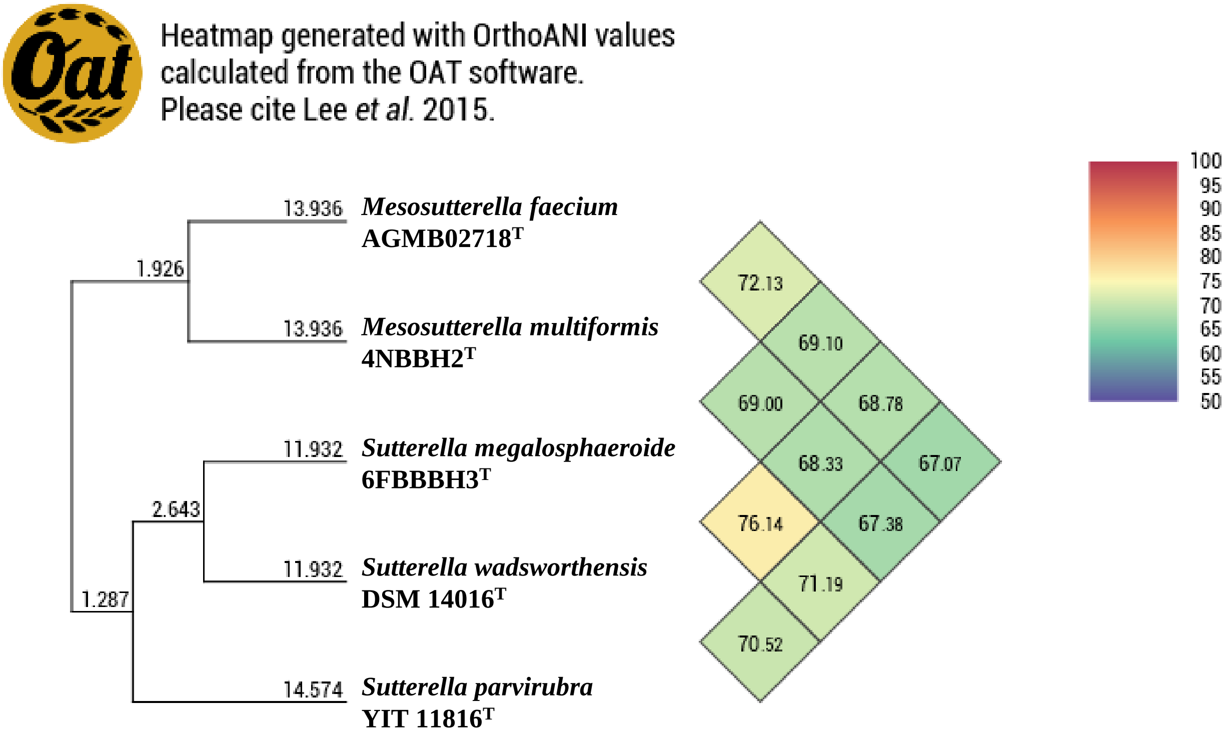

Supplementary Figure S4. Heatmap generated with OrthoANI. ANI value calculated using the OAT software, comparing strain AGMB02718^{T} and closely related strains.

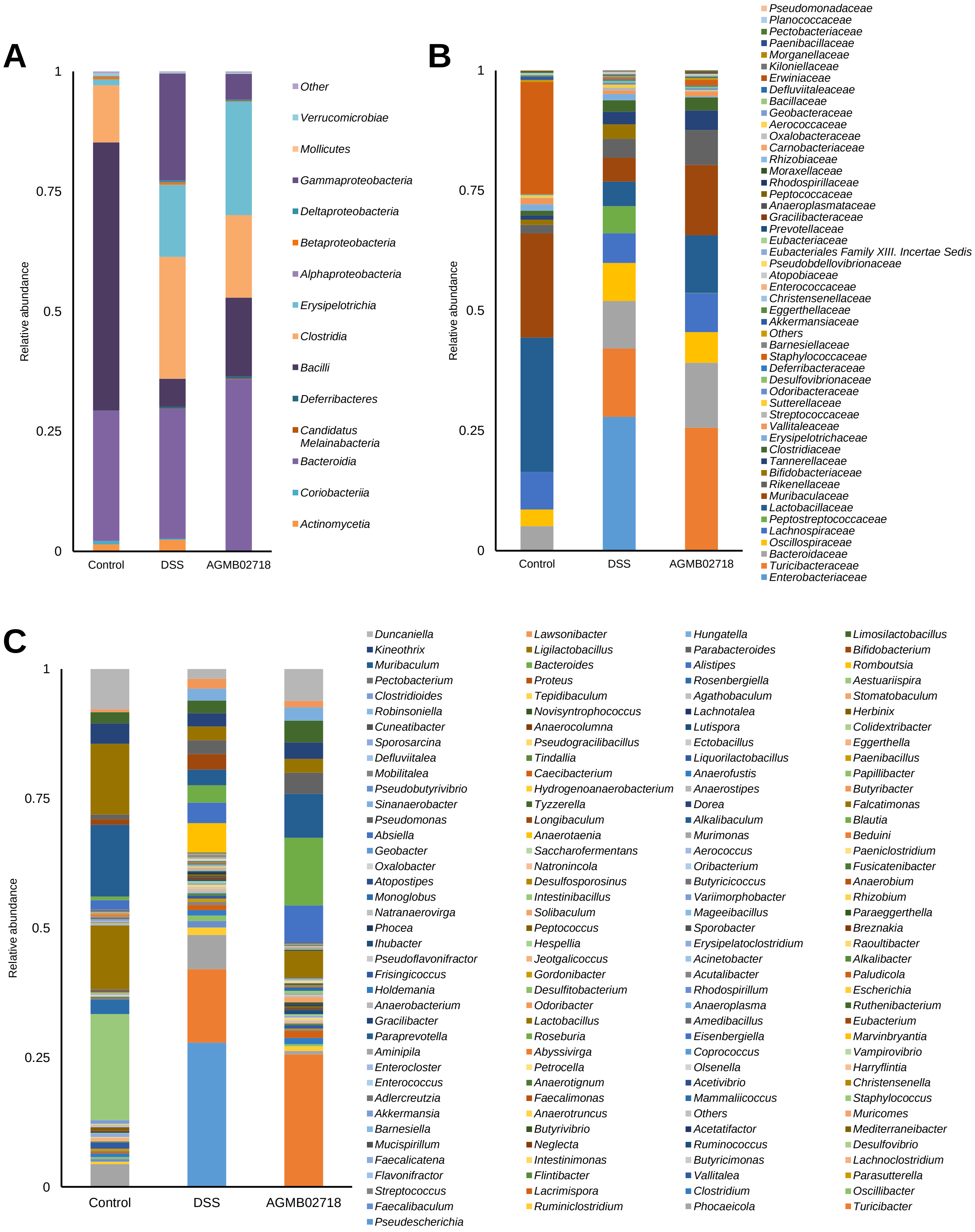

Supplementary Figure S5. Relative abundance of taxa at the **(A)** class, **(B)** family, and **(C)** genus levels among the three groups.

Supplementary Figure

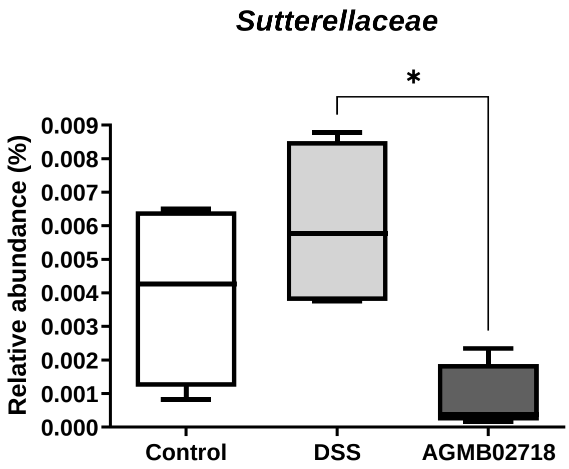

Supplementary Figure S6. Relative abundance of *Sutterellaceae* ratio among three groups. Data are shown as mean ± SEM (*n*=*5*). *P<0.05 by One-way ANOVA with Tukey's multiple comparisons.
